# Concordance analysis of methylation biomarkers detection in self-collected and physician-collected samples in cervical neoplasm

**DOI:** 10.1186/s12885-015-1411-x

**Published:** 2015-05-19

**Authors:** Cheng-Chang Chang, Rui-Lan Huang, Yu-Ping Liao, Po-Hsuan Su, Yaw-Wen Hsu, Hui-Chen Wang, Chau-Yang Tien, Mu-Hsien Yu, Ya-Wen Lin, Hung-Cheng Lai

**Affiliations:** 1 Department of Obstetrics and Gynecology, Tri-Service General Hospital, National Defense Medical Center, Taipei, Taiwan Republic of China; 2 Graduate Institute of Medical Sciences, National Defense Medical Center, Taipei, Taiwan Republic of China; 3 Department of Obstetrics and Gynecology, Shuang Ho Hospital, Taipei Medical University, Taipei, Taiwan Republic of China; 4 Laboratory of Epigenetics and Cancer Stem Cells, National Defense Medical Center, Taipei, Taiwan Republic of China; 5 Graduate Institute of Life Sciences, National Defense Medical Center, Taipei, Taiwan Republic of China; 6 Department and Graduate Institute of Microbiology and Immunology, National Defense Medical Center, Taipei, Taiwan Republic of China; 7 Department of Obstetrics and Gynecology, School of Medicine, College of Medicine, Taipei Medical University, Taipei, Taiwan Republic of China

**Keywords:** Cervical cancer, DNA methylation, Biomarker, Self-collected, Physician-collected, Real-time quantitative methylation-specific polymerase chain reaction (QMSP)

## Abstract

**Background:**

Non-attendance at gynecological clinics is a major limitation of cervical cancer screening and self-collection of samples may improve this situation. Although HPV testing of self-collected vaginal samples is acceptable, the specificity is inadequate. The current focus is increasing self-collection of vaginal samples to minimize clinic visits. In this study, we analyzed the concordance and clinical performance of DNA methylation biomarker (*PAX1*, *SOX1*, and *ZNF582*) detection in self-collected vaginal samples and physician-collected cervical samples for the identification of cervical neoplasm.

**Methods:**

We enrolled 136 cases with paired methylation data identified from abnormal Pap smears (n = 126) and normal controls (n = 10) regardless of HPV status at gynecological clinics. The study group comprised 37 cervical intraepithelial neoplasm I (CIN1), 23 cervical intraepithelial neoplasm II (CIN2), 16 cervical intraepithelial neoplasm III (CIN3), 30 carcinoma *in situ* (CIS), 13 squamous cell carcinomas (SCCs) and seven adenocarcinomas (ACs)/adenosquamous carcinomas (ASCs). *PAX1*, *SOX1* and *ZNF582* methylation in study samples was assessed by real-time quantitative methylation-specific polymerase chain reaction analysis. We generated methylation index cutoff values for the detection of CIN3+ in physician-collected cervical samples for analysis of the self-collected group. Concordance between the physician-collected and self-collected groups was evaluated by Cohen’s Kappa. Sensitivity, specificity and area under curve (AUC) were calculated for detection of CIN3+ lesions. Finally, we produced an optimal cutoff value with the best sensitivity from the self-collected groups.

**Results:**

We generated a methylation index cutoff value from physician-collected samples for detection of CIN3+. There were no significant differences in sensitivity, specificity of *PAX1*, *SOX1* and *ZNF582* between the self-collected and physician-collected groups. The methylation status of all three genes in the normal control samples, and the CIN 1, CIN2, CIN3, CIS, ACs/ASCs and SCC samples showed reasonable to good concordance between the two groups (κ = 0.443, 0.427, and 0.609 for *PAX1*, *SOX1*, and *ZNF582*, respectively). In determining the optimal cutoff values from the self-collected group, *ZNF582* showed the highest sensitivity (0.77; 95%CI, 0.65–0.87) using a cutoff value of 0.0204.

**Conclusions:**

Methylation biomarker analysis of the three genes for detection of CIN3+ lesions shows reasonable to good concordance between the self-collected and physician-collected samples. Therefore, self-collection of samples could be adopted to decrease non-attendance and improve cervical screening.

## Background

Cervical cancer remains one of the main causes of death from cancer among women worldwide [1]. Cytology-based screening has successfully reduced mortality associated with cervical cancer [2]. However, the majority of cases of cervical cancer are still associated with absent or deficient screening. In previous studies, approximately 50 % of cervical cancers were diagnosed in women who were not screened [3, 4]. Complete participation would achieve a greater improvement in screening effectiveness than intensifying screening policies [3]. Therefore, it is important to improve participation rates among women with a history of non-attendance.

Epidemiological studies have emphasized that human papillomaviruses (HPVs) are the main etiological factor for cervical cancer and that these viruses are present in almost all cervical cancer tissues [5]. Screening participation rates for cervical cancer can be improved by offering non-attending women the tools to collect a vaginal sample at home. Self-collection is an acceptable method to potentially increase participation [6]. Studies have demonstrated that self-collected samples are suitable for HPV DNA testing and can increase participation rates in primary screening for cervical cancer [6–11]. However, women whose self-collected specimens test positive for high-risk HPV (hrHPV) require additional triage testing because the specificity of assays for hrHPV is insufficient to justify direct referral for colposcopy in all cases [11, 12]. Although cytology is an accepted and standard method of examination in triage for hrHPV-positive women [13], cytological testing of self-collected samples does not yield reliable results and a visit to a physician is required [14].

In normal, precancerous and cervical cancer tissues, the DNA methylation profiles of the host genome may indicate tissue-specific perturbations that occur during carcinogenesis [15]. DNA methylation leaves a heritable record of such interactions and is an ideal biomarker for cancer detection [16–20], which could be used to triage possible cases of cervical cancer [21–24]. Previously, we used a CpG island microarray approach to identify novel genes that were silenced by methylation in cervical squamous cell carcinoma (SCC) [25]. Quantitative analysis of the *PAX1* and *SOX1* genes can be used effectively for detection of cases of CIN that are grade 3 or worse (CIN3+) [17]. Using methylated DNA sequence immunoprecipitation coupled with microarray analysis to identify other genes with clinical applications, we found that the gene for zinc finger protein 582 (ZNF582) was highly methylated in SCC [18]. This gene is also highly methylated in adenocarcinoma (AC) of the cervix [26]. In Taiwanese Gynecologic Oncology Group (TGOG) studies, we used a methylation biomarker and hrHPV tests to detect CIN3+ lesions in low grade squamous intraepithelial lesions (LSIL). ZNF582 methylation is implicated as a promising biomarker for use in the positive triage of cytological diagnoses of low grade squamous intraepithelial lesions [27]. Combined parallel testing using Pap smears and PAX1 or SOX1 methylation tests may provide better performance than a combination of Pap smears with HPV-testing in detection of cervical neoplasm [28]. The clinical performance of *PAX1*, *SOX1,* and *ZNF582* as biomarkers of cervical neoplasm has been validated in multi-center clinical trials; therefore, analysis of changes in the methylation status of these genes could be applied for self-collected vaginal samples.

The aim of this study was to validate the concordance and clinical performance of *PAX1*, *SOX1*, and *ZNF582* methylation for detection of CIN3+ lesions in self-collected and physician-collected vaginal samples. Our hope is that these genes will serve as sensitive methylation biomarkers for clinical cervical cancer screening of self-collected samples.

## Methods

### Patients and sampling

**Fig. 1:**
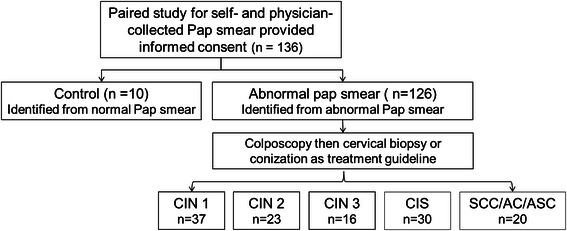
The study design flowchart. All women used a cytobrush (CooperSurgical, CT, USA) to collect a vaginal sample as instructed by a physician. Subsequently, a physician-collected cervical sample was obtained. Patients with abnormal Pap smear results were managed by colposcopy followed by cervical biopsy or conization according to treatment guidelines. (CIN: cervical intraepithelial neoplasm, CIS: carcinoma *in situ*, SCC: squamous cell carcinoma, AC: adenocarcinoma, ASC: adenosquamous carcinoma)

The flowchart illustrating the study design is shown in Fig. 1. We randomly selected a sample set from women attending our gynecologic outpatient department. All women used a cytobrush (CooperSurgical, CT, USA) to collect a vaginal sample as instructed by a physician. Subsequently, a physician-collected cervical sample was obtained.

Patients with normal cervixes (n = 10), and those with CIN1 (n = 37), CIN2 (n = 23), CIN3 (n = 16), carcinoma *in situ* (CIS) (n = 30), SCC (n = 13), and adenocarcinoma (AC)/adenosquamous carcinoma (ASC) (n = 7) of the uterine cervix participated in this study. Patients whose cervical samples had normal cytology served as control subjects. The patients were diagnosed, treated, and had their tissues banked at the National Defense Medical Center, Taipei, Taiwan as described [29]. All CINs and invasive cancers were confirmed by histopathology. Control patients were recruited from healthy women who underwent routine Pap screening during the same period. Exclusion criteria included pregnancy, chronic or acute systemic viral infections, a history of cervical neoplasm, skin or genital warts, an immunocompromised state, presence of other cancers, and previous surgery of the uterine cervix.

### Generation of methylation index cutoff values and clinical accuracy calculation

Real-time quantitative methylation-specific polymerase chain reaction (QMSP) was performed to assess the methylation status of *PAX1*, *SOX1* and *ZNF582* in study samples. We generated methylation index cutoff values from physician-collected cervical samples for detection of CIN3+ in self-collected samples. Sensitivity, specificity and area under curve (AUC) were calculated for detection of CIN3+ lesions. Finally, we produced an optimal cutoff value with the best sensitivity from the self-collected groups.

### Real-time quantitative methylation-specific polymerase chain reaction amplification of DNA

Genomic DNA was extracted from the collected specimens using a protocol established for tissue banking. The concentration of DNA was determined using the Nanodrop 1000 (Thermo Fisher Scientific). QMSP was performed after bisulfite treatment of denatured genomic DNA. Gene symbols, primers, and probes for QMSP are available on request [17]. The *COL2A* gene was used as an internal reference to adjust the amount of input DNA by amplifying non-CpG sequences in each sample. QMSP was performed with a TaqMan probe system in a Roche LightCycler® 480 system. The 5′ and 3′ ends of the probes were labeled with 6-carboxy-fluorescein (6-FAM) and a quencher dye, respectively. The 20 μL reaction mix contained 2 μL of bisulfite template DNA (2 μL), primers (250 nM each), TaqMan probe (225 nM), and FastStart Universal Probe Master (10 μL) (ROX, Roche). For the TaqMan-based QMSP, each sample was analyzed in duplicate. The reactions were performed by using an initial incubation at 95 °C for 10 min, followed by 45 cycles of 95 °C for 15 s and annealing and extension at the appropriate temperatures for 1 min. The level of DNA methylation was described as the methylation index (M-index) and calculated as follows: 10,000 × 2^[(Cp of *COL2A*) – (Cp of gene)]^ [18]. The QMSP was deemed to be a failure if the Cp value of *COL2A* was higher than 36.

### Ethics statement

Informed consent to participation in this study was obtained from all patients and control subjects. This study was conducted in accordance with the guidelines, and with the approval of, the Ethics Committee of the Institutional Review Boards of the Tri-Service General Hospital, National Defense Medical Center (TSGHNDMCIRB-096-05-090).

### Statistical analysis

Data were analyzed using the updated MedCalc version 14. To determine the detection rate, CIN3 was taken as a cutoff value for the QMSP analysis of three genes in self-collected and physician-collected samples. The receiver operating characteristic (ROC) curve was used to select the optimal cutoff value according to the maxima of sensitivity and specificity to distinguish CIN3+ (including CIN3, CIS, SCC, AC and ASC) and CIN2^−^ (including normal controls, CIN1 and CIN2) patients [30]. The McNemar test was used to test the proportion of self-collected and physician-collected samples in CIN3+ groups by the optimal cutoff value of physician-collected. The concordance between the self-collected and physician-collected samples was measured by Cohen’s Kappa coefficient. Differences with *P*-values less than 0.05 were considered to indicate statistical significance.

## Results

### Population, study design flowchart and cytology/histology of study samples

We invited 136 women to participate in this study of self-collected samples with paired methylation data from samples of 10 normal control cervixes, 37 CIN1, 23 CIN2, 16 CIN3, 30 CIS, and 20 invasive cancers (13 SCCs and 7 ACs/ASCs) (Fig. 1). Details of the cytology/histology and mean age of the subjects are shown in Table 1.

**Table 1:** Cytology/histology and mean age of study participants

Diagnosis	Cases	Mean age ± SD
	(n)	(years)
Total	136	47.9 ± 12.9
Normal cytology	10	53.0 ± 16.2
CIN1	37	43.3 ± 11.3
CIN2	23	47.1 ± 14.3
CIN3	16	48.1 ± 12.5
CIS	30	48.0 ± 11.9
SCC	13	54.2 ± 11.6
AC/ASC	7	57.1 ± 14.9

*SD* Standard deviation, *CIN1* cervical intraepithelial neoplasm type 1, *CIN2* cervical intraepithelial neoplasm type 2, *CIN3* cervical intraepithelial neoplasm type 3, *CIS* carcinoma *in situ*, *SCC* squamous cervical carcinoma, *AC* adenocarcinoma, *ASC* adenosquamous carcinoma

### Validation of clinical performance and concordance analysis of methylation biomarkers in self-collected and physician-collected samples

**Fig. 2:**
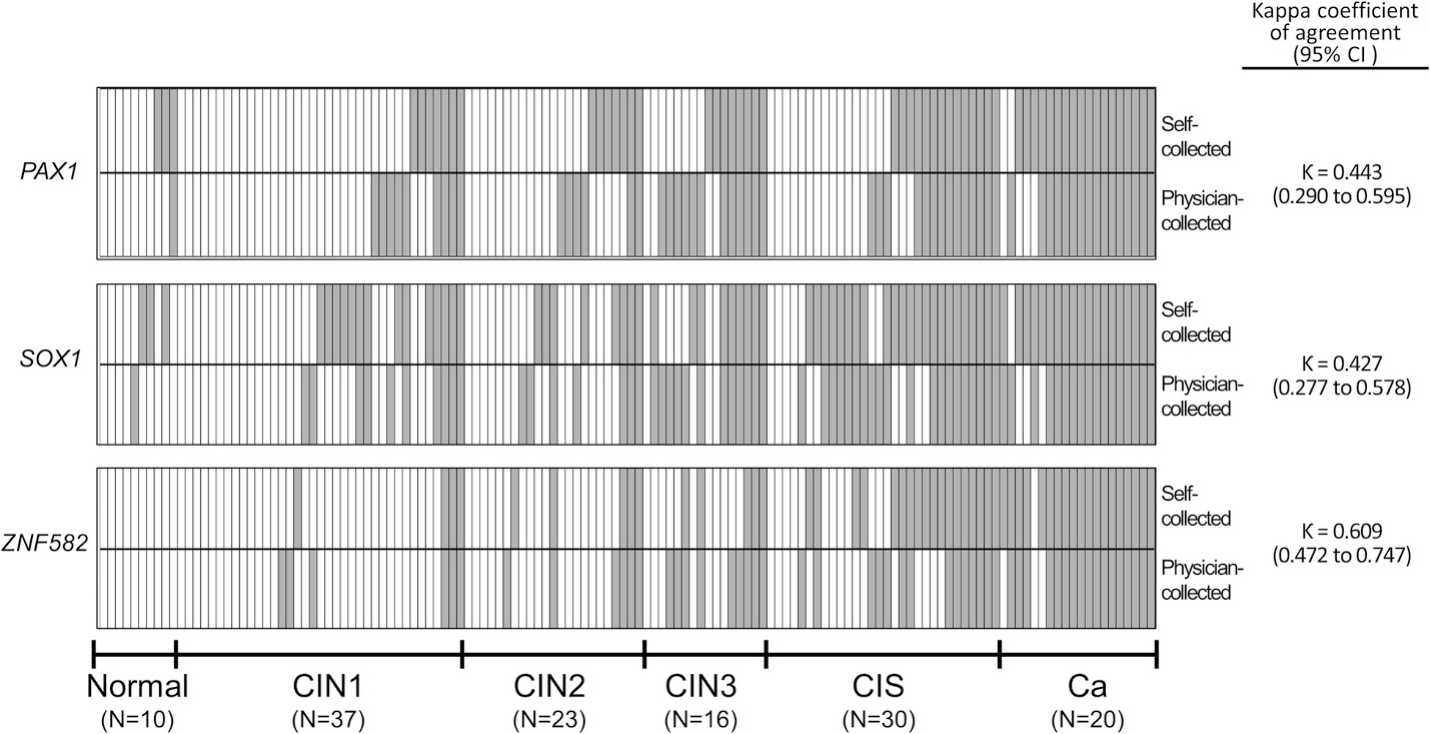
Heat map of study patients in self-collected and physician-collected samples. Women with normal cervixes (n = 10), and those with CIN1 (n = 37), CIN2 (n = 23), CIN3 (n = 16), carcinoma *in situ* (CIS) (n = 30), SCC (n = 13), and adenocarcinoma (AC)/adenosquamous carcinoma (ASC) (n = 7) of the uterine cervix participated in this study. The methylation status of all three genes are shown: dark, high methylation and light, low methylation

We generated a methylation index cutoff value from physician-collected samples for detection of CIN3+ and then compared the methylation of *PAX1*, *SOX1* and *ZNF582* genes in physician-collected and self-collected samples. There were no significant differences in the sensitivity and specificity of the QMSP analysis of *PAX1*, *SOX1*, and *ZNF582* between the self-collected and physician-collected samples (Table 2). In addition, the *PAX1*, *SOX1*, and *ZNF582* methylation profiles in the CIN3+ positive cases among the self-collected samples were similar to those among the physician-collected samples (percentage positive among self-collected samples: 60.6 %, 77.3 %, and 63.6 %, respectively; percentage positive among physician-collected samples: 64.64 %, 74.24 %, and 60.61 %, respectively) (*P* = 0.81, 0.81, and 0.81, respectively; Table 2). Self-collection was found to be comparable with physician-collected samples for the detection of cervical methylation biomarkers (κ = 0.443, 0.427, and 0.609 for *PAX1*, *SOX1*, and *ZNF582*, respectively; Fig. 2).

**Table 2:** Comparison the detection of CIN3^+^ between the physician-collected and self-collected samples using methylation of PAX1, SOX1 and ZNF582 genes

Physician-collected						Self-collected				
	Cutoff Point^a^	Positive case of CIN2-	Positive case of CIN3+	Sensitivity (95 % CI)	Specificity (95 % CI)	Positive in CIN2-	Positive in CIN3+	Sensitivity (95 % CI)	Specificity (95 % CI)	*P* ^*b*^
		(Total, N = 70)	(Total, N = 66)			(Total N = 70)	(Total N = 66)			
PAX 1	0.014	16	42	0.64	0.77	17	40	0.61	0.76	0.81
		(22.86 %)	(64.64 %)	(0.51 to 0.75)	(0.66 to 0.86)	(24.3 %)	(60.6 %)	(0.48 to 0.72)	(0.64 to 0.85)	
SOX1	0.156	18	49	0.74	0.74	25	51	0.77	0.64	0.81
		(25.71 %)	(74.24 %)	(0.62 to 0.84)	(0.62 to 0.84)	(35.7 %)	(77.3 %)	(0.65 to 0.87)	(0.52 to 0.75)	
ZNF582	0.214	12	40	0.61	0.83	9	42	0.64	0.87	0.81
		(17.14 %)	(60.61 %)	(0.48 to 0.72)	(0.72 to 0.91)	(12.9 %)	(63.6 %)	(0.51 to 0.75)	(0.77 to 0.94)	
Any of SOX1, PAX1		25	53	0.80	0.64	32	53	0.80	0.54	1.00
		(35.71 %)	(80.3 %)	(0.69 to 0.89)	(0.52 to 0.75)	(44.7 %)	(80.3 %)	(0.69 to 0.89)	(0.42 to 0.66)	
Any of SOX1, ZNF582		22	53	0.80	0.69	27	53	0.80	0.61	1.00
		(31.4 %)	(80.3 %)	(0.69 to 0.89)	(0.56 to 0.79)	(38.6 %)	(80.3 %)	(0.69 to 0.89)	(0.49 to 0.73)	
Any of PAX1, ZNF582		23	48	0.73	0.67	20	48	0.73	0.71	1.00
		(32.9 %)	(72.7 %)	(0.60 to 0.83)	(0.55 to 0.78)	(28.6 %)	(72.7 %)	(0.60 to 0.83)	(0.59 to 0.82)	
Any one of three		29	57	0.86	0.59	34	55	0.83	0.51	0.75
		(41.4 %)	(86.4 %)	(0.76 to 0.94)	(0.46 to 0.70)	(48.6 %)	(83.3 %)	(0.72 to 0.91)	(0.39 to 0.64)	
Any two of three		12	40	0.71	0.83	11	44	0.67	0.84	0.52
		(17.1 %)	(60.6 %)	(0.48 to 0.72)	(0.72 to 0.91)	(15.7 %)	(66.7 %)	(0.5 to 0.78)	(0.74 to 0.92)	

*CI* confident interval

^a^The optimal cutoff value of methylation index is identified in the physician-collected group and testing in the self-collected group

^b^Performed a McNemar test for the comparison of proportions of CIN3+ using the cutoff value of physician-collected samples

### Optimization of the clinical accuracy of methylation biomarkers using the cutoff values of the self-collected group

**Fig. 3:**
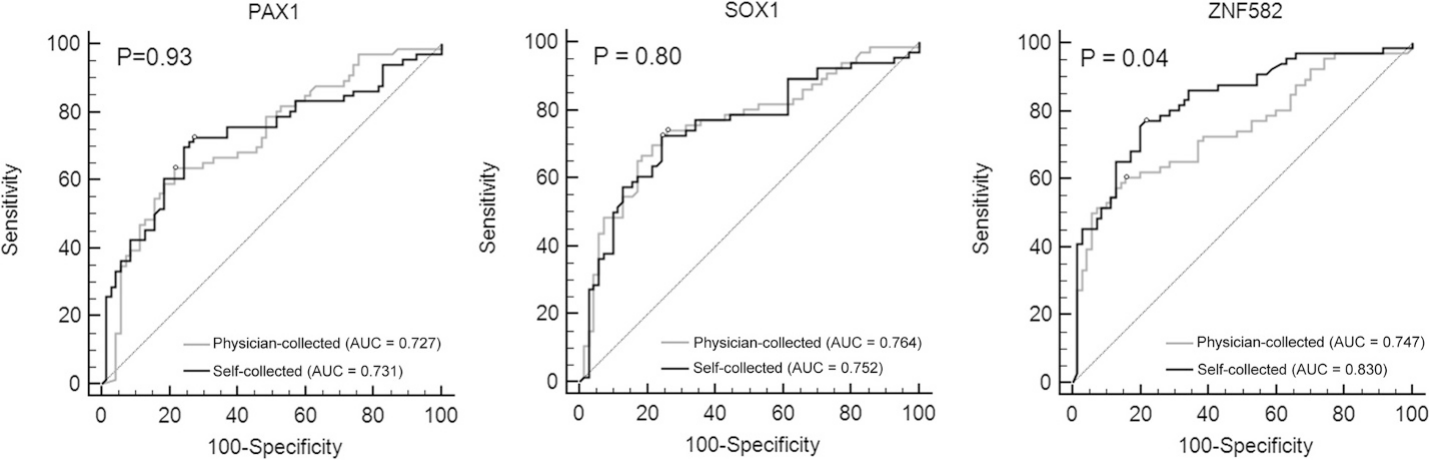
Concordance in the clinical performance of methylation biomarkers between the self-collected and physician-collected groups. Receiver operating characteristic (ROC) curve analysis of *PAX1*, *SOX1* and *ZNF582*. The area under curve (AUC) of the ROC curve of each gene was calculated for the diagnosis of CIN3 and further progressed (CIN3+) lesions. Differences with *P*-values less than 0.05 were considered to indicate statistical significance

The clinical performance of QMSP of *ZNF582* in the self-collected group was better than that in the physician-collected group using a cutoff value generated from the physician-collected group (sensitivity: 0.64; 95%CI, 0.51–0.75 vs. 0.61 95%CI, 0.48–0.72, specificity: 0.87; 95%CI, 0.77–0.94 vs 0.83; 95%CI, 0.72–0.91; Table 2). Using a ROC curve to obtain the best cutoff values from the self-collected group, we found that QMSP of *ZNF582* had the highest sensitivity (0.77; 95%CI, 0.65–0.87) and specificity (0.77; 95%CI, 0.66–0.86) (Table 3) at a cutoff value of 0.0204. There were no differences in the AUCs of *PAX1* and *SOX1* between the self-collected and physician-collected groups (0.731 vs. 0.727, *P* = 0.93 and 0.752 vs. 0.764, *P* = 0.80, respectively, Fig. 3). The AUC of *ZNF582* in the self-collected group showed significantly better clinical performance than that in the physician-collected group (0.830 vs. 0.747, *P* = 0.04; Fig. 3).

**Table 3:** The optional cutoff value generated from self-collected samples for detection of CIN3^+^ in self-collected samples with methylation of PAX1, SOX1 and ZNF582 genes

	Cutoff Point^a^	Positive cases of CIN2-	Positive cases of CIN3+	Sensitivity (95 % CI)	Specificity (95 % CI)
		(Total, N = 70)	(Total, N = 66)		
PAX1	0.0027	20	48	0.73	0.71
		(28.6 %)	(70.6 %)	(0.60 to 0.83)	(0.60 to 0.82)
SOX1	0.516	18	48	0.73	0.74
		(25.7 %)	(72.7 %)	(0.60 to 0.83)	(0.62 to 0.84)
ZNF582	0.0204	16	51	0.77	0.77
		(22.9 %)	(77.3 %)	(0.65 to 0.87)	(0.66 to 0.86
Any of SOX1, PAX1		28	57	0.86	0.60
		(38.9 %)	(86.3 %)	(0.76 to 0.94)	(0.48 to 0.72)
Any of SOX1, ZNF582		26	55	0.83	0.63
		(36.1 %)	(83.4 %)	(0.72 to 0.91)	(0.51 to 0.74)
Any of PAX1, ZNF582		28	59	0.89	0.60
		(38.9 %)	(89.4 %)	(0.79 to 0.96)	(0.48 to 0.72)
Any one of three		35	62	0.94	0.50
		(50.0 %)	(93.9 %)	(0.85 to 0.98)	(0.38 to 0.62)
Any two three		12	47	0.71	0.83
		(17.1 %)	(71.2 %)	(0.59 to 0.82)	(0.72 to 0.91)

*CIN2* (including normal, CIN 1 and CIN 2), *CIN3*^*+*^ including CIN3, CIS, SCC, ASC and AC), *CI* confident interval

^a^The optimal cutoff value of methylation index is identified in the self-collected group

## Discussion

Our data showed reasonable to good concordance in the DNA methylation the *PAX1*, *SOX1*, and *ZNF582* genes for detection of CIN3+ lesions between self-collected vaginal samples and physician-collected cervical samples. These findings indicate that in the future, the requirement for patients to visit a physician for screening could be reduced by submitting self-collected samples. Compared with the current cytology-based call-recall programs, self-collected vaginal samples can increase access to cervical screening and may help to further reduce the incidence of cervical cancer by increasing the rate of participation in screening programs [2, 11].

Our study for the comparison of the concordance of methylation status between self-collected vaginal samples and physician-collected cervical samples was conducted in a relatively large sample-size. Self-collection was found to be comparable with physician-collected samples for the detection of cervical methylation biomarkers. This is consistent with other recent reports, which have also shown high concordance in the results of methylation analysis of self-sampled vaginal material and physician-collected cervical scrapes [31]. Furthermore, our study was conducted using a relatively large number of high-grade squamous intraepithelial neoplasm (HSIL, including CIN2, CIN3/CIS, SCC, AC/ASC) samples, including seven cases of ACs/ASCs. In addition, assessment of the methylation biomarkers in *ZNF582* provided the best clinical accuracy among self-collected samples (AUC: 0.83; sensitivity, 0.77 (95%CI, 0.65–0.87); specificity, 0.77 (95%CI, 0.66–0.86), using a cutoff of 0.0204). These results are comparable with those of well-performed cytological testing, indicating that methylation biomarker analysis of self-collected vaginal samples has the potential for use in population-based studies comparing the clinical performance of cytological testing for alternative methods of screening of cervical cancer.

The limitation of this study is the restricted sample set, especially in the normal controls, because we focused on investigating the concordance between physician-collected samples and self-collected samples obtained using a cytobrush. This device was designed for physician sampling and is not particularly practical for use in self-collection sampling. Consequently, the level of compliance and success in obtaining a sample using this method was low among the women in the control group and few samples were obtained. In addition, this was a hospital-based study and so the results may not be representative of the general population. A standardized education program and user-friendly tools for self-collection are also warranted.

In developed countries with extensive infrastructure for conducting cytological examinations, Pap smears combined with methylation tests may perform better than a combination of Pap smears with HPV-testing in the detection of cervical neoplasm [28]. The reasons underlying the lack of participation in screening programs among women in developed countries are complex. Some examples of the barriers that have been reported are practical, such as appointment times and embarrassment [32]; therefore, creative and sensitive methods that take into consideration these barriers to participation in cervical cancer screening are required [32]. Self-collected sampling is time-saving and avoids embarrassment. For a non-attendee, self-collection of samples for molecular screening of hrHPV could be a suitable method for primary cervical cancer screening followed by cytology-based triage. Although the detection rate for CIN2+ or CIN3+ lesions is promising, cervical cytology sampling still requires intervention by a clinician [21, 23, 24]. Recent studies have used methylation biomarkers to triage patients who screened positive for hrHPV. The sensitivity of direct triage by combined analysis of the promoter methylation of miR-124-2 and the MAL genes in self-collected cervicovaginal material was similar to that of triage with cytological analysis of an additional physician-collected smear [20]. The search for complete methylation markers for use in triage of hrHPV-positive women or in primary screening of cervical cancer alone may further revolutionize cervical cancer screening.

Although self-collection of samples for hrHPV testing is an acceptable method screening for hrHPV infection, insufficient specificity will lead false-positive results in many patients in the absence of cervical neoplasm. Triage of these patients is required to confirm a true cervical intraepithelial neoplasm. Given the lack of infrastructure for conducting cytological examinations in low-resource areas, cytological screening is not ideal for triage; furthermore, the sensitivity of this method varies from 30 % to 87 % [33]. In contrast, only a few neoplastic cells are required for the detection of promoter methylation within a gene of interest using the QMSP assay. We determined that the highest sensitivity values for the detection of CIN3+ lesions by determining the methylation status of *PAX1*, *SOX1*, and *ZNF582* in self-collected samples was 0.73, 0.73, and 0.77, respectively (Table 3). The clinical performance of this type of assay resembled that of a traditional cytological examination. The potential use of these new biomarkers as tools for cervical cancer screening as well as their possible use in the developing world to triage hrHPV-positive women during primary screening warrants further validation.

We used CIN3+ rather than CIN2+ as the cutoff in our study because of the equivocal nature of CIN2 lesions when diagnosed and the heterogeneity of their DNA methylation profiles [17, 34]. While only 5 % of CIN2 lesions progress to invasive cancer and approximately 40 % regress, the corresponding percentages for CIN3 lesions are 33 % and 12 %, respectively [35]. The pathology of CIN2 lesions is not clearly defined and these are the most difficult for pathologists to confirm among all Pap smear diagnoses [36, 37]. The clinical management of patients with CIN2 lesions should be reassessed using the most accurate techniques. The incorporation of molecular markers, such as DNA methylation profiles, into cervical cancer screening might help to decrease the number of unnecessary referrals and repeat diagnostic procedures, which are not only a drain on financial resources but also inflict an unnecessary burden on the patient. Additional studies are required to define the nature of CIN2 lesions with or without DNA methylation in longitudinal studies.

## Conclusions

Our data confirm the reasonable to good concordance between DNA methylation biomarker profiles analyzed in self-collected and physician-collected samples for detection of CIN3+ lesions. This indicates that cervical cancer screening could be carried out not only on samples collected by physicians in a clinic setting, but also on self-collected vaginal samples. To confirm our results, the performance of our assay should be evaluated in prospective population-based clinical trials.

## Abbreviations

*PAX1*: Paired box 1 gene
*SOX1*: Sex determining region Y-box 1
*ZNF582*: Zinc finger protein 582
CIN1: Cervical intraepithelial neoplasm I
CIN2: Cervical intraepithelial neoplasm II
CIN3: Cervical intraepithelial neoplasm III
CIS: Carcinoma *in situ*
SCCs: Squamous cell carcinomas
ACs: Adenocarcinomas
ASCs: Adenosquamous carcinomas
AUC: Area under curve
QMSP: Quantitative methylation-specific polymerase chain reaction
HPV: Human papillomavirus
hrHPV: High-risk HPV

## References

[1] Forouzanfar MH, Foreman KJ, Delossantos AM, Lozano R, Lopez AD, Murray CJ (2011). Breast and cervical cancer in 187 countries between 1980 and 2010: a systematic analysis. Lancet.

[2] Peto J, Gilham C, Fletcher O, Matthews FE (2004). The cervical cancer epidemic that screening has prevented in the UK. Lancet.

[3] Bos AB, Rebolj M, Habbema JD, van Ballegooijen M (2006). Nonattendance is still the main limitation for the effectiveness of screening for cervical cancer in the Netherlands. Int J Cancer.

[4] de Bie RP, Vergers-Spooren HC, Massuger LF, Siebers AG, der Pol MR S-v, Vedder JE (2011). Patients with cervical cancer: why did screening not prevent these cases?. Am J Obstet Gynecol.

[5] Zur Hausen H (2002). Papillomaviruses and cancer: from basic studies to clinical application. Nat Rev Cancer.

[6] Bosgraaf RP, Verhoef VM, Massuger LF, Siebers AG, Bulten J, de Kuyper-de Ridder GM (2014). Comparative performance of novel self-sampling methods in detecting high-risk human papillomavirus in 30,130 women not attending cervical screening. Int J Cancer J Int du cancer.

[7] Lazcano-Ponce E, Lorincz AT, Cruz-Valdez A, Salmeron J, Uribe P, Velasco-Mondragon E (2011). Self-collection of vaginal specimens for human papillomavirus testing in cervical cancer prevention (MARCH): a community-based randomised controlled trial. Lancet.

[8] Virtanen A, Nieminen P, Luostarinen T, Anttila A (2011). Self-sample HPV tests as an intervention for nonattendees of cervical cancer screening in Finland: a randomized trial. Cancer Epidemiol Biomarkers Prev.

[9] Zhao FH, Lewkowitz AK, Chen F, Lin MJ, Hu SY, Zhang X (2012). Pooled analysis of a self-sampling HPV DNA test as a cervical cancer primary screening method. J Natl Cancer Inst.

[10] Szarewski A, Cadman L, Mesher D, Austin J, Ashdown-Barr L, Edwards R (2011). HPV self-sampling as an alternative strategy in non-attenders for cervical screening - a randomised controlled trial. Br J Cancer.

[11] Snijders PJ, Verhoef VM, Arbyn M, Ogilvie G, Minozzi S, Banzi R (2013). High-risk HPV testing on self-sampled versus clinician-collected specimens: a review on the clinical accuracy and impact on population attendance in cervical cancer screening. Int J Cancer.

[12] Cuzick J, Clavel C, Petry KU, Meijer CJ, Hoyer H, Ratnam S (2006). Overview of the European and North American studies on HPV testing in primary cervical cancer screening. Int J Cancer.

[13] Rijkaart DC, Berkhof J, Rozendaal L, van Kemenade FJ, Bulkmans NW, Heideman DA (2012). Human papillomavirus testing for the detection of high-grade cervical intraepithelial neoplasia and cancer: final results of the POBASCAM randomised controlled trial. Lancet Oncol.

[14] Garcia F, Barker B, Santos C, Brown EM, Nuno T, Giuliano A (2003). Cross-sectional study of patient- and physician-collected cervical cytology and human papillomavirus. Obstet Gynecol.

[15] Farkas SA, Milutin-Gasperov N, Grce M, Nilsson TK (2013). Genome-wide DNA methylation assay reveals novel candidate biomarker genes in cervical cancer. Epigenetics.

[16] Wentzensen N, Sherman ME, Schiffman M, Wang SS (2009). Utility of methylation markers in cervical cancer early detection: appraisal of the state-of-the-science. Gynecol Oncol.

[17] Lai HC, Lin YW, Huang RL, Chung MT, Wang HC, Liao YP (2010). Quantitative DNA methylation analysis detects cervical intraepithelial neoplasms type 3 and worse. Cancer.

[18] Huang RL, Chang CC, Su PH, Chen YC, Liao YP, Wang HC (2012). Methylomic analysis identifies frequent DNA methylation of zinc finger protein 582 (ZNF582) in cervical neoplasms. PLoS One.

[19] Sova P, Feng Q, Geiss G, Wood T, Strauss R, Rudolf V (2006). Discovery of novel methylation biomarkers in cervical carcinoma by global demethylation and microarray analysis. Cancer Epidemiol Biomarkers Prev.

[20] Verhoef VM, Bosgraaf RP, van Kemenade FJ, Rozendaal L, Heideman DA, Hesselink AT (2014). Triage by methylation-marker testing versus cytology in women who test HPV-positive on self-collected cervicovaginal specimens (PROHTECT-3): a randomised controlled non-inferiority trial. Lancet Oncol.

[21] Hesselink AT, Heideman DA, Steenbergen RD, Coupe VM, Overmeer RM, Rijkaart D (2011). Combined promoter methylation analysis of CADM1 and MAL: an objective triage tool for high-risk human papillomavirus DNA-positive women. Clin Cancer Res.

[22] Overmeer RM, Louwers JA, Meijer CJ, van Kemenade FJ, Hesselink AT, Daalmeijer NF (2011). Combined CADM1 and MAL promoter methylation analysis to detect (pre-)malignant cervical lesions in high-risk HPV-positive women. Int J Cancer.

[23] Hansel A, Steinbach D, Greinke C, Schmitz M, Eiselt J, Scheungraber C (2014). A promising DNA methylation signature for the triage of high-risk human papillomavirus DNA-positive women. PLoS One.

[24] Eijsink JJ, Lendvai A, Deregowski V, Klip HG, Verpooten G, Dehaspe L (2012). A four-gene methylation marker panel as triage test in high-risk human papillomavirus positive patients. Int J Cancer.

[25] Lai HC, Lin YW, Huang TH, Yan P, Huang RL, Wang HC (2008). Identification of novel DNA methylation markers in cervical cancer. Int J Cancer.

[26] Chang CC, Huang RL, Wang HC, Liao YP, Yu MH, Lai HC (2014). High methylation rate of LMX1A, NKX6-1, PAX1, PTPRR, SOX1, and ZNF582 genes in cervical adenocarcinoma. Int J Gynecol Cancer.

[27] Lin H, Chen TC, Chang TC, Cheng YM, Chen CH, Chu TY (2014). Methylated ZNF582 gene as a marker for triage of women with Pap smear reporting low-grade squamous intraepithelial lesions - a Taiwanese Gynecologic Oncology Group (TGOG) study. Gynecol Oncol.

[28] Lai HC, Ou YC, Chen TC, Huang HJ, Cheng YM, Chen CH (2014). PAX1/SOX1 DNA methylation and cervical neoplasia detection: a Taiwanese Gynecologic Oncology Group (TGOG) study. Cancer medicine.

[29] Chu TY, Hwang KS, Yu MH, Lee HS, Lai HC, Liu JY (2002). A research-based tumor tissue bank of gynecologic oncology: characteristics of nucleic acids extracted from normal and tumor tissues from different sites. Int J Gynecol Cancer.

[30] Youden WJ (1950). Index for rating diagnostic tests. Cancer.

[31] Boers A, Bosgraaf RP, van Leeuwen RW, Schuuring E, Heideman DA, Massuger LF (2014). DNA methylation analysis in self-sampled brush material as a triage test in hrHPV-positive women. Br J Cancer.

[32] Waller J, Jackowska M, Marlow L, Wardle J (2012). Exploring age differences in reasons for nonattendance for cervical screening: a qualitative study. BJOG.

[33] Nanda K, McCrory DC, Myers ER, Bastian LA, Hasselblad V, Hickey JD (2000). Accuracy of the Papanicolaou test in screening for and follow-up of cervical cytologic abnormalities: a systematic review. Ann Intern Med.

[34] Holowaty P, Miller AB, Rohan T, To T (1999). Natural history of dysplasia of the uterine cervix. J Natl Cancer Inst.

[35] Ostor AG (1993). Natural history of cervical intraepithelial neoplasia: a critical review. Int J Gynecol Pathol.

[36] Stoler MH, Schiffman M (2001). Atypical Squamous Cells of Undetermined Significance-Low-grade Squamous Intraepithelial Lesion Triage Study G: Interobserver reproducibility of cervical cytologic and histologic interpretations: realistic estimates from the ASCUS-LSIL Triage Study. Jama.

[37] Carreon JD, Sherman ME, Guillen D, Solomon D, Herrero R, Jeronimo J (2007). CIN2 is a much less reproducible and less valid diagnosis than CIN3: results from a histological review of population-based cervical samples. Int J Gynecol Pathol.

